# Polar Cryoconite Associated Microbiota Is Dominated by Hemispheric Specialist Genera

**DOI:** 10.3389/fmicb.2021.738451

**Published:** 2021-11-25

**Authors:** Jasmin L. Millar, Elizabeth A. Bagshaw, Arwyn Edwards, Ewa A. Poniecka, Anne D. Jungblut

**Affiliations:** ^1^School of Earth and Environmental Sciences, Cardiff University, Cardiff, United Kingdom; ^2^Department of Life Sciences, The Natural History Museum, London, United Kingdom; ^3^Institute of Biological, Environmental and Rural Sciences, Aberystwyth University, Ceredigion, United Kingdom

**Keywords:** cryoconite, illumina sequencing, Antarctic microbiology, Arctic microbiology, pole-to-pole, 16S rRNA gene, 18S rRNA gene

## Abstract

Cryoconite holes, supraglacial depressions containing water and microbe-mineral aggregates, are known to be hotspots of microbial diversity on glacial surfaces. Cryoconite holes form in a variety of locations and conditions, which impacts both their structure and the community that inhabits them. Using high-throughput 16S and 18S rRNA gene sequencing, we have investigated the communities of a wide range of cryoconite holes from 15 locations across the Arctic and Antarctic. Around 24 bacterial and 11 eukaryotic first-rank phyla were observed in total. The various biotic niches (grazer, predator, photoautotroph, and chemotroph), are filled in every location. Significantly, there is a clear divide between the bacterial and microalgal communities of the Arctic and that of the Antarctic. We were able to determine the groups contributing to this difference and the family and genus level. Both polar regions contain a “core group” of bacteria that are present in the majority of cryoconite holes and each contribute >1% of total amplicon sequence variant (ASV) abundance. Whilst both groups contain Microbacteriaceae, the remaining members are specific to the core group of each polar region. Additionally, the microalgal communities of Arctic cryoconite holes are dominated by *Chlamydomonas* whereas the Antarctic cryoconite holes are dominated by *Pleurastrum*. Therefore cryoconite holes may be a global feature of glacier landscapes, but they are inhabited by regionally distinct microbial communities. Our results are consistent with the notion that cryoconite microbiomes are adapted to differing conditions within the cryosphere.

## Introduction

Cold climate habitats are present across the globe, sustaining a surprising abundance of life above, within, and below the ice (Boetius et al., 2015). Approximately 10% of the Earth’s surface is covered by glacial ice (Hornberger and Winter, 2009), and glaciers and ice sheets are now regarded as a distinct biome (Hodson et al., 2008; Anesio and Laybourn-Parry, 2012). On the surface of glaciers and ice sheets, particular hotspots of diversity occur when these living cells and the associated organic matter they produce accumulate. The term “cryoconite” refers to the microbially-aggregated wind-blown dust, organic and mineral matter that forms on glacial surfaces, particularly in the ablation zone (Cook et al., 2015). The dark colour of the cryoconite depresses ice surface albedo, resulting in the localised melting of surface ice, often forming near-cylindrical holes containing meltwater in ice surfaces known as cryoconite holes (Nordenskjold, 1875). Cryoconite provides a habitat in ice for a plethora of organisms (Vincent and Laybourn-Parry, 2009; Cameron et al., 2012; Lutz et al., 2015), and is common to glaciers worldwide, including those of the Arctic and Antarctic (Cook et al., 2015). Many of the microbes present produce extracellular polymeric substances that allow them to form biofilms and increase the habitability of their surrounding environment. In cryoconite holes, these polymeric substances cause other materials such as sediment to adhere to the cells (Langford et al., 2010), forming a stabilised habitat (Langford et al., 2010; Takeuchi et al., 2010; Webster-Brown et al., 2015; Cook et al., 2016).

The structure of cryoconite holes varies according to the prevailing physical conditions. In cold, dry climates such as continental Antarctica, cryoconite holes are usually covered by an ice lid through most or all of the year (Tranter et al., 2004; Webster-Brown et al., 2015). In the McMurdo Dry Valleys, ice lids of up to 30cm have been observed on the majority of cryoconite holes year-round (Fountain et al., 2004; Tranter et al., 2004). Some of these thick lids may melt during particularly warm periods, years apart (Foreman et al., 2007). Around 50% of lidded holes are hydrologically connected under the ice surface; the other 50% are completely isolated (Fountain et al., 2004). The holes may melt under the ice lid, connecting to one another on a seasonal or sub-seasonal timescale (Bagshaw et al., 2007). These closed holes are a contrasting environment to the seasonally “open” cryoconite holes found elsewhere, particularly in many Arctic and mountainous regions (Cook et al., 2015). Open holes do not have a permanent ice lid and can hence exchange gas with the atmosphere. These holes primarily occur on glacial ablation zones that exhibit seasonal melting (Bagshaw et al., 2012), experiencing regular flushing by stream flow which distributes cryoconite across the glacier surface (Irvine-Fynn et al., 2010).

Varying cryoconite environments lead to a range of cryoconite microbial ecosystems (Edwards et al., 2011). Sequences extracted from cryoconite communities tend to be dominated by Proteobacteria, Bacteroidetes, Cyanobacteria, and microalgae (Cameron et al., 2012; Edwards et al., 2013; Sommers et al., 2018). They also harbour fungi, protists, and micro-animals (meiofauna; Zawierucha et al., 2015). The relative abundance of these organisms varies with biogeography (Liu et al., 2017; Darcy et al., 2018). Research to date has revealed that communities are more similar within glaciers than between glaciers, but lack sufficient detail to determine whether trends are local, regional, or global. In a study using community fingerprinting and clone library analysis, it was discovered that there is a divide between the bacterial communities of the Arctic and Antarctic (Cameron et al., 2012). These results raised important questions about the potentially distinct biomes of the poles and the variability of cryoconite. However, the methods used could only yield limited detail in comparison to insights arising from the rapid evolution of high throughput DNA sequencing technologies in the ensuing decade. It has not yet been established which taxa are contributing to this difference in community composition.

In this study, we used 16S and 18S rRNA gene high throughput sequencing to cover Archaea, Bacteria, and Eukarya. Through these methods, we show the presence of distinct communities of the Arctic and Antarctic in significantly more detail than previous studies (Mueller and Pollard, 2004; Cameron et al., 2012; Hell et al., 2013; Sommers et al., 2018). We also reveal a genus-level breakdown of the composition of cryoconite communities. The breadth of geographic coverage in our sample set allowed comparison not only between the polar regions, but also between different environments within each polar region. We compare Greenland cryoconite from cryoconite holes formed the year of collection, and “ice core” cryoconite frozen the previous year. We also compare samples from ice sheet interiors with marginal locations. Together, these results provide an in-depth analysis of cryoconite ecology at a global scale, and improve understanding of the distinct communities of the polar regions.

## Materials and Methods

### Cryoconite Sampling Sites and Sample Collection

**Figure 1:**
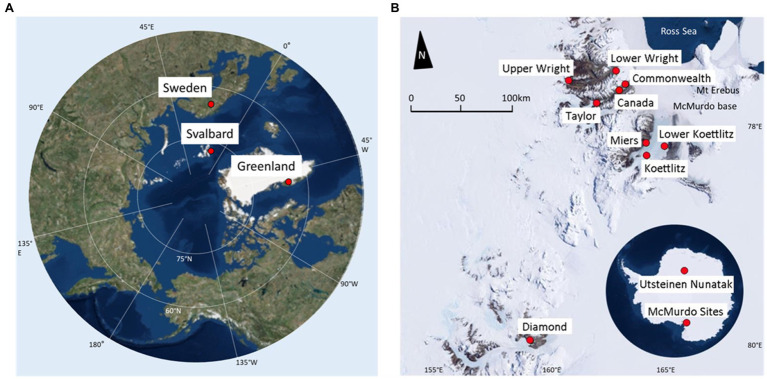
Sampling locations. **(A)** Arctic map, highlighting regions sampled. Points marked indicate sample site locations and names. **(B)** Map of Antarctic sampling locations. Antarctic continental map highlighting the Utsteinen Scoop and the McMurdo Dry Valleys. Underlying is a McMurdo Valleys map, which shows a detailed view of those sites. Base maps were created using ArcGIS® software by Esri. ArcGIS® and ArcMap™ are the intellectual property of Esri and are used herein under license. Source: ArcWorld Supplement.

Samples were collected from 85 individual cryoconite holes across 15 sites (Table 1). Ten locations were in the Antarctic and five were in the Arctic, and all were collected during the regional summer melt season (Figure 1). The Antarctic samples were collected in the McMurdo Dry Valleys, with the exception of the Utsteinen samples, which were collected in Queen Maud Land near the Utsteinen Nunatak (Lutz et al., 2019). Collection technique was determined by the presence and thickness of ice lid or ice layers above. Three sample types were collected from the southwest of Greenland. Those labelled “Greenland Margin” were collected within 2km of the ice edge in 2014. Samples labelled “Greenland Core” were frozen cryoconite layers collected from shallow subsurface layers of 1m ice cores on inland ice in the “Dark Zone,” at Camp Black and Bloom (Poniecka et al., 2019; Williamson et al., 2020). Typically the layers are located around 10–30cm depth of the core and are believed to be retained from the previous year’s cryoconite holes following burial under frozen snowpack (Nicholes et al., 2019). Greenland core samples were drilled in 2015 using a hand auger and the sediments separated from the meltwater (Poniecka, 2020). Greenland surface samples were also collected at Camp Black and Bloom 2016. Two sample sets were collected from elsewhere in the Arctic at Storglaciaren in Sweden (2017), and Midtre Lovénbreen in Svalbard (2016). Exposed cryoconite was sampled using a syringe or spoon and stored in clean, sterile Whirlpak bags or tubes, and frozen until analysis (Edwards et al., 2011; Poniecka et al., 2019; Poniecka, 2020). Antarctic Dry Valley samples from Canada, Taylor and Commonwealth glaciers were collected from the base of frozen cores (20–50cm deep) drilled from cryoconite holes between 2005 and 2009 (Bagshaw et al., 2012). The cores were melted at room temperature, the meltwater removed, and the sediment transferred to Nalgene bottles previously rinsed six times with deionised water, then refrozen at −20°C. The cryoconite from Koettlitz, Wright and Darwin Glaciers were collected using a sterile spatula as described in Webster-Brown et al. (2015). Utsteinen Nunatak samples were accessed in 2017 using a Kovacs drill, and the sediment removed using a sterilised scoop (Lutz et al., 2019). Greenland margin and ‘Kangerlussuaq’ samples were collected from melted cryoconite holes in summer 2012 and 2014, where clean nitrile gloves were used to scoop sediment into Ziploc bags previously rinsed with deionised water. Samples were frozen within a few hours of collection. All samples were transported frozen to home laboratories and remained frozen at −20°C until analysis.

**Table 1:** GPS locations and site names for cryoconite samples from the Arctic and Antarctic.

Sample site	Location	No. of samples	Width (cm)	Ice Lid
Canada Glacier, Antarctica	77.6°S, 163.0°E	9	23–57	Present
Commonwealth Glacier, Antarctica	77.6°S, 163.3°E	9	28–64	Present
Taylor Glacier, Antarctica	77.7°S, 162.0°E	9	41–52	Present
Upper Wright Glacier, Antarctica	77.5°S, 162.9°E	7	37–57	Present
Lower Wright Glacier, Antarctica	77.5°S, 160.7°E	6	27–43	Present
Diamond Glacier, Antarctica	79.8°S, 159.0°E	4	41–52	Present
Miers Glacier, Antarctica	78.8°S, 163.7°E	5	14–39	Present
Upper Koettlitz Glacier, Antarctica	78.3°S, 163.63°E	9	28–97	Present
Lower Koettlitz Glacier, Antarctica	78.1°S, 164.2°E	5	35–55	Present
Utsteinen Scoop, Antarctica	72.0°S, 23.3°E	2	5–10	Present
Kangerlussuaq, Greenland	67.1°N, 50.7°E	3	15–40	Absent
Ice Margin, Greenland	67.2°N, 50.0°E	4	5–99	Absent
Ice Core, Greenland	67.1°N, 50.7°E	3	1–15	Present (layers of ice above)
Storglaciären, Sweden	67.1°N, 18.6°E	3	Unknown	Absent
Midtre Lovénbreen, Svalbard	78.8°N, 12.1°E	5	1–15	Absent

### DNA Extraction, PCR, Purification, Quantification, and Illumina Miseq Sequencing

Subsamples of each cryoconite sample were melted at 4°C. Genomic DNA was extracted using the DNeasy PowerSoil Kit (QIAGEN) according to manufacturer’s instructions. The prokaryotic 16S (V4 region) and eukaryotic 18S (V9 region) rRNA genes were amplified by PCR. DNA volumes of 0.5, 1.0, and 1.5μl DNA were added to 19μl of GoTaq Polymerase reaction mix. In cases where the DNA concentration was too low to yield detectable quantities of PCR product, volumes of 1.5, 2.0, and 2.5μl were used. The final master mix contained 4μl 5x GoTaq Flexi buffer, 2μl MgCl2 (25μM, Promega, Madison, United States), 0.8μl Bovine Serum Albumin (BSA, 20mg/ml BSA, NEB, United Kingdom), 0.16μl of 200μM dnTPs (Bioline, United Kingdom), 9.84μl H2O, 0.2μl Taq polymerase (5U/μl, Promega, Madison, United States), and 1μl of each forward and reverse primer (10μM). The forward primer 515 F (GTGCCAGCMGCCGCGGTAA) and reverse primer 806R (GGACTACHVGGGTWTCTAAT) containing the MiSeq sequencing adapters and 12-nucleotide Golay barcodes were used to amplify the V4 hyper-variable region of the bacteria and archaea 16S rRNA gene (260bp, Caporaso et al., 2011). The primers 1391F and EukBr were used to amplify the V9 hypervariable region of the eukaryote 18S rRNA genes containing MiSeq sequencing adapters, a 12-nucleotide Golay barcode on the reverse primer (130bp, Amaral-Zettler et al., 2009; Caporaso et al., 2011, 2012). The 16S rRNA gene was amplified in an thermocycler using the following program: initial denaturation at 94°C for 2min followed by 30cycles of denaturation at 94°C for 45s, annealing at 50°C for 60s, and elongation at 72°C for 90s; and then, a final extension of 72°C for 10min. The 18S rRNA gene was amplified using the following program: initial denaturation at 94°C for 3min followed by 35cycles of denaturation at 94°C for 45s, annealing at 57°C for 60s, and elongation at 72°C for 90s; then, a final extension of 72°C for 10min. The PCR products along with a negative control were verified by gel electrophoresis using 1.5% agarose for 18S rRNA gene and 1% agarose gel for 16S rRNA gene PCR-products. Following purification according to AxyPrep Mag PCR clean-up protocol (Axygen), the triplicate PCR-products per sample were combined and concentrations determined using a Qubit 2.0 Fluorometer (ThermoFisher Scientific, Waltham, MA, United States) and the manufacturer’s protocol. The 16S and 18S rRNA gene amplicons of each sample were separately in preparation for sequencing. The PCR products were sequenced at the Natural History Museum sequencing facility using an Illumina MiSeq platform (Illumina, San Diego, CA, United States).

### 16S and 18S rRNA Gene Sequence Analysis

The raw sequence data were processed using QIIME2 v2018.8 (Caporaso et al., 2012; Bolyen et al., 2019). Sequences were demultiplexed based on Golay barcodes as a pre-processing step on the Illumina Miseq platform. Reads were quality-filtered, joined, chimeras were removed, and amplicon sequence variants (ASVs) were generated using DADA2 (Callahan et al., 2016). Alignment was performed with MAFFT (Katoh and Standley, 2013), and low complexity and repeating sequences were removed using the mask function in QIIME2. Phylogenetic trees were constructed with Fasttree (Price et al., 2009). Taxonomy was assigned with sklearn-based taxonomy classifier using the SILVA 138 database (Quast et al., 2013). Representative sequences for each ASV were assigned to the highest confidence and identity match on the SILVA 138 database. ASVs assigned to the same taxon were grouped for relative abundance analyses. Chloroplast and mitochondrial DNA were excluded from the prokaryote dataset. ASVs with a frequency<3 were removed and the dataset was rarefied to 13,044 16S rRNA gene sequences and 6,261 18S rRNA gene sequences. Relative taxa abundance and ASV counts were then generated using QIIME2. About 0.04% of the 16S rRNA gene assignment output, and 12.62% of 18S rRNA gene assignment output were unassigned to a domain or any lower classification. These were removed as they are unlikely to be relevant and correct sequences. The highest Genbank BLASTn match was also obtained for top most abundant 20 16S rRNA gene features and 20 18S rRNA gene features for verification. Alpha diversity was calculated using both Shannon’s diversity (Shannon, 1948), and the Simpson index (Simpson, 1949). Beta diversity was tested using R packages vegan and phyloseq, and ggplot2 was used to visualise the results (McMurdie and Holmes, 2013; Wickham, 2016; Oksanen et al., 2019). Non-metric dimensional scaling of ASV relative abundance was performed using Bray-Curtis distances. The species richness correlation was calculated using Pearson’s test for correlation. Occupancy of ASVs in each sample was plotted based on mean relative abundance of each ASV per sample against presence of ASV in samples using ggplot2 (Wickham, 2016).

## Results

### Bacteria and Archaea Composition and Community Assembly in Arctic and Antarctic Cryoconite

**Figure 2:**
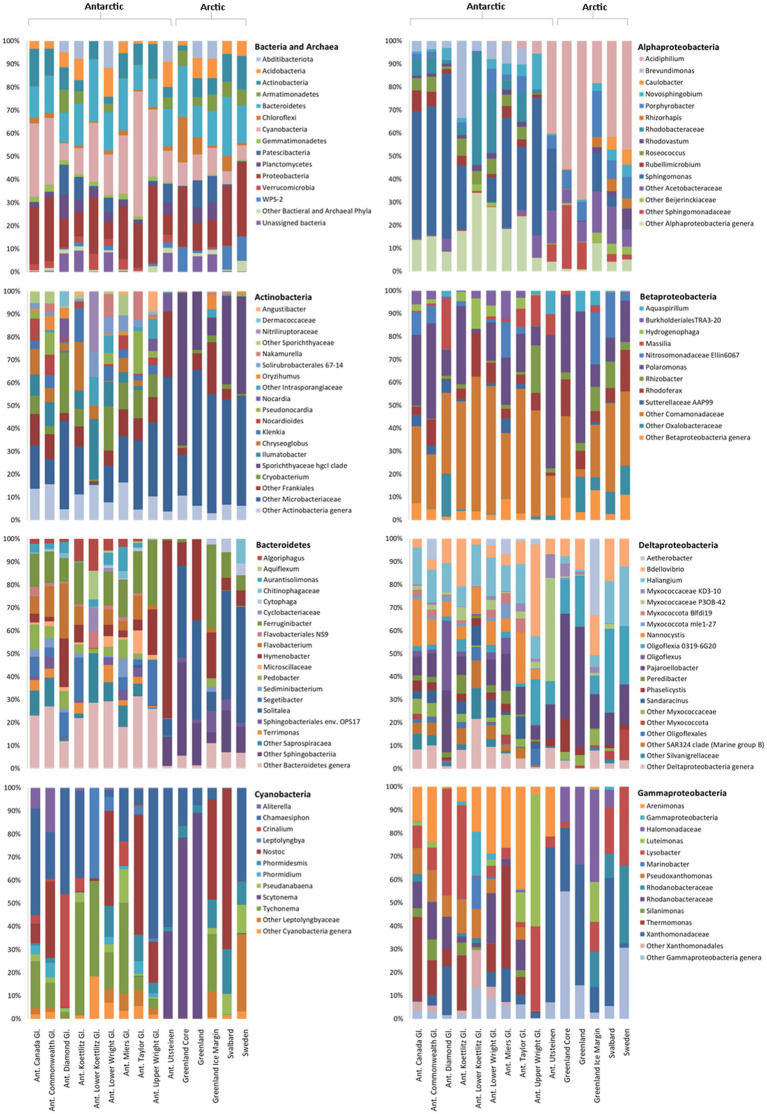
Relative abundance of bacteria in Arctic and Antarctic cryoconite, averaged by glacier. Top left: Bacterial phyla. Phyla contributing to <1% of the total abundance are grouped as “Other.” Remaining panels: Relative abundance of genera within the top four most abundant phyla. Proteobacteria have been divided into alpha-, beta-, delta-, and gammaproteobacteria. Genera contributing to <1% of the total abundance are grouped as “Other.” Where a genus was unknown, lowest rank known is shown. Taxa were assigned according to the SILVA database. “Ant. Gl.” denotes Antarctic Glaciers.

Following filtering and rarefaction, 13,044 16S rRNA gene sequences per sample were obtained and 24 bacterial and one archaeal phyla were identified. About 4,497 distinct 16S rRNA gene ASVs were identified, 313 of which were shared between the Arctic and Antarctic. Only nine archaeal sequences were found, appearing in seven samples in the McMurdo Dry Valleys. Cyanobacteria, Proteobacteria, Bacteroidetes, and Actinobacteria were the most abundant bacterial phyla, accounting for 65% of the total 16S rRNA gene ASVs (Figure 2). These phyla were present across all samples. All other phyla contributed to <6% of the total 16S rRNA gene ASVs.

**Figure 3:**
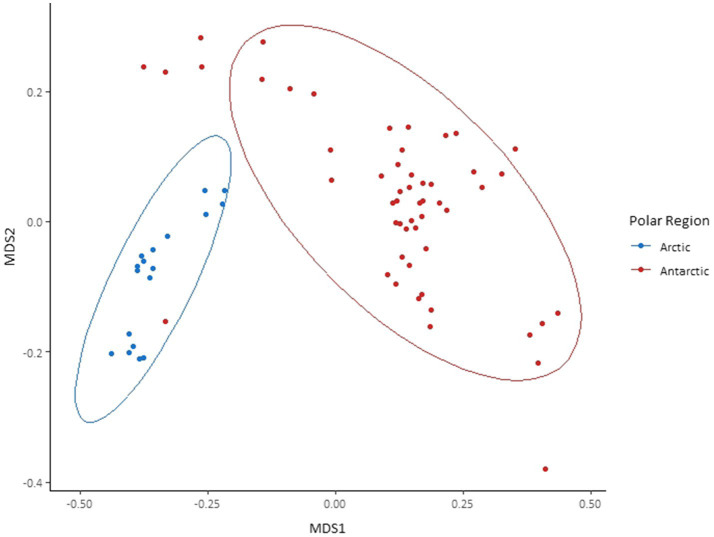
Bray-Curtis dissimilarity of 16S rRNA gene amplicon sequence variants (ASVs) found in each cryoconite and visualised by non-metric multidimensional scaling (NMDS) ordination. Cryoconite holes are grouped by polar region. Ellipses represent 95% CIs.

**Figure 4:**
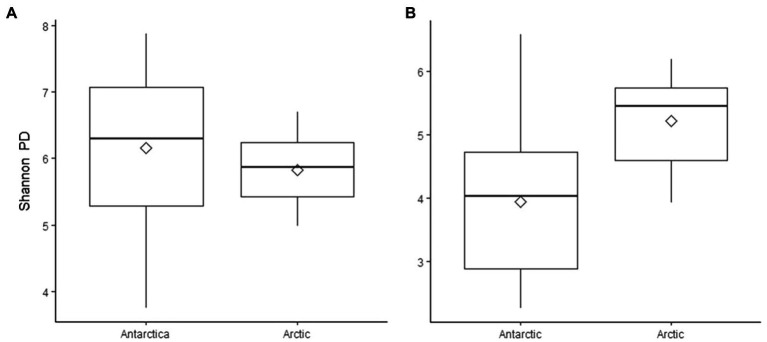
Shannon’s phylogenetic diversity of **(A)** 16S rRNA gene ASVs and **(B)** 18S rRNA gene ASVs in cryoconite samples, grouped by polar region. Diamonds indicate mean values.

Although the same phyla had the highest relative abundance across all locations, there are noticeable differences between the Arctic and Antarctic 16S rRNA gene cryoconite composition. Arctic cryoconite communities had a higher relative abundance of Chloroflexi and Armatimonadetes, and a lower relative abundance of Cyanobacteria. Cyanobacteria accounted for 9% of ASVs across Arctic locations compared to 24% in cryoconite from Antarctica. The variation between Arctic and Antarctic taxonomic diversity within the datasets was explored by analysis of beta diversity. We found a compositional divide between Arctic and Antarctic cryoconite ecosystems based on the relative abundance of taxa. An ANOSIM test of the dissimilarity between poles using the Bray-Curtis method produced an *R* value of 0.723 at *p*=0.0001 (pairwise community dissimilarity), suggesting a significant level of dissimilarity (Supplementary Table 2). Non-metric multi-dimensional scaling of Bray-Curtis distances ordinates this separation between Arctic and Antarctic cryoconite communities (Figure 3). The Greenland margin and ice core samples cluster closest with the other Greenland samples, and then other Arctic sites. While there was more variation between the types of Greenland samples (margin, ice core, and ice sheet surface) than within groups, none were outliers within the grouping of Arctic samples. Therefore, Greenland margin and Greenland ice core samples have been grouped with the other Arctic samples during polar region comparisons. The range and mean number of distinct ASVs was considerably lower in the Arctic than Antarctic (Supplementary Table 1). Alpha diversity and evenness of bacteria and archaea in the samples was investigated further using the Shannon diversity indices (Figure 4A). The lowest richness was identified in cryoconite samples from the Antarctic Upper Wright (4.76) and Lower Wright (4.85) glaciers. The highest values were also from the Antarctic such the Commonwealth (7.15) and Canada (6.66) glaciers. The mean values were 6.22 for the Antarctic and 5.62 for the Arctic.

The four most abundant bacterial phyla were investigated further to elucidate the composition of their genera and contribution to the dissimilarity between the poles (Figure 2). In the Actinobacteria, there is a striking difference between Arctic and Antarctic samples. While the Antarctic samples, with the exception of the Upper Wright glacier, contain a diverse array of Actinobacteria, the Arctic samples are dominated by Sporichthyaceae hgcl clade and Microbacteriaceae which make up 77% of Arctic Actinobacteria sequences. In the Antarctic samples, the Sporichthyaceae hgcl clade contributes 0.04% and the Microbacteriaceae contributes 27%. The psychrophile *Cryobacterium* contributes to 9% of total Actinobacteria sequences and 12% of Antarctic Actinobacteria sequences. In the Bacteroidetes, the Arctic cryoconite holes vary from the Antarctic cryoconite holes in their high relative abundance of *Solitalea* (39% of Bacteroidetes sequences in the Arctic compared to 1% in the Antarctic). The Bacteroidetes ASVs cover 155 genera. Around 139 of these were present in Antarctic and only 51 in Arctic cryoconite communities. Of these 37 present in Arctic samples, 11were only present in the Greenland margin site. These included the *Segetibacter* and *Spirosoma*. The most abundant Cyanobacterial genera were *Tychonema*, *Chamaesiphon*, *Nostoc*, *Scytonema*, and *Tychonema*. Together these contributed to 71% of the total cyanobacterial ASVs. All other groups each contributed <6%. Around 28 genera were found in total, all of which were present in the Antarctic cryoconite communities, but only 11 of these were found in the Arctic samples. The largest difference in percentage proportion between the Arctic and Antarctic was *Scytonema* (42% of Arctic and 3% of Antarctic samples). This genus is absent from the Greenland margin sediment but dominated the other Greenland samples. It was also absent in cryoconite from Svalbard.

The Alphaproteobacteria made up 38% of Proteobacteria 16rRNA gene sequences in the Antarctic and 66% of Proteobacteria sequences in Arctic cryoconite, the Betaproteobacteria comprised 33% of Antarctic and 18% of Arctic Proteobacteria sequences, and the Gammaproteobacteria comprised 22% of Antarctic and 6% of Arctic Proteobacteria sequences. Other 16S rRNA gene sequences belonged to Deltaproteobacteria (7 and 10% of Proteobacteria 16S rRNA gene sequences in Antarctic and Arctic cryoconite, respectively) and unassigned Proteobacteria (0.02 and 0.05% of Proteobacteria 16S rRNA gene sequences in the Antarctic and Arctic cryoconite, respectively). The composition of Betaproteobacteria genera was similar between the Arctic and Antarctic, but there were more differences between the polar regions in the Alphaproteobacteria, Deltaproteobacteria, and Gammaproteobacteria. In the Alphaproteobacteria, *Sphingomonas* had a high relative abundance in the Antarctic (41% of Alphaproteobacteria compared to 3% in the Arctic) whereas *Acidiphilium* had higher relative abundant in the Arctic (50% of Alphaproteobacteria compared to 7% in the Antarctic). The genus *Nannocystis*, which contributed 10% to the Antarctic Deltaproteobacteria, was absent from the Arctic samples. Oligoflexia 0319-6G20 contributed to 4% of Antarctic Deltaproteobacteria sequences and 4% of Arctic Deltaproteobacteria. Of the 31 genera that contributed to >1% of Deltaproteobacterial 16S rRNA gene sequences, 28 were present in the Antarctic samples and nine in the Arctic samples. A large proportion of the 16S rRNA gene sequences assigned as Gammaproteobacteria in the Arctic was assigned to the Halomonadaceae (21%), a group of halophiles, and Xanthomonadaceae (24%). Interestingly, the Gammaproteobacteria 16S rRNA gene composition in samples from the Greenland margin samples bore a greater similarity to the Svalbard samples than the other Greenland samples, largely consisting of, *Lysobacter* and unassigned Rhodanobacteraceae in addition to Halomonadaceae and Xanthomonadaceae. About 49 of the 51 Gammaproteobacteria genera were found in the Antarctic, 17 were present in the Arctic, and 15 were present in both the polar regions.

**Figure 5:**
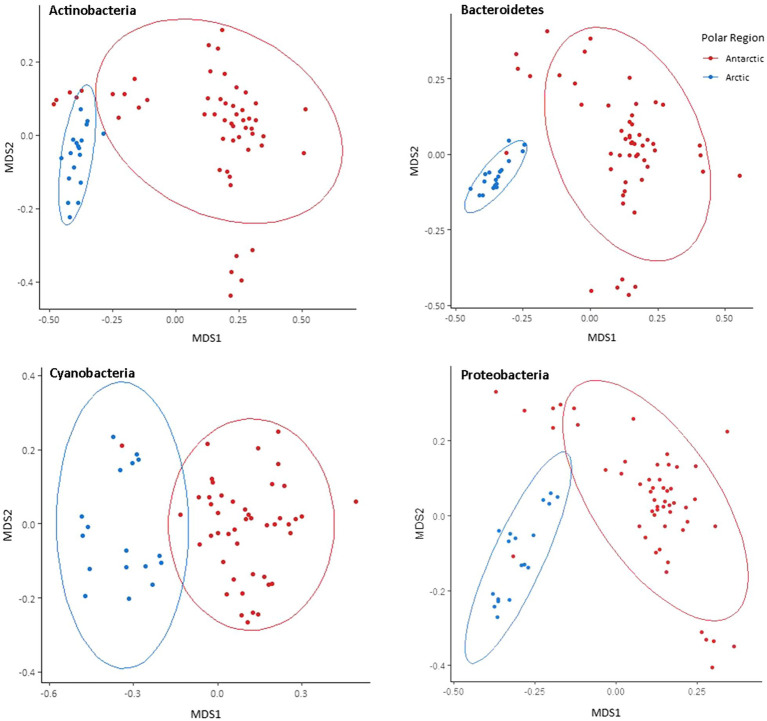
Bray-Curtis dissimilarity of Arctic and Antarctic 16S rRNA gene ASVs in the most abundant four bacterial phyla. Visualised by NMDS ordination. Holes are grouped by polar region. Ellipses represent 95% CIs.

Separate non-metric multidimensional scaling (NMDS) analysis of the four most abundant phyla Cyanobacteria, Bacteroides, Proteobacteria, and Actinobacteria 16S rRNA gene sequences suggested that the presence of distinct communities in the Arctic and Antarctica (Figure 5). ANOSIM analyses produce *R* values of 0.5 or above for Actinobacteria and Proteobacteria at a significance level of *p*=0.0001. However, the *R* values are greater when testing for differences in phyla composition between locations (0.24–0.66 between poles, 0.24–0.75 between locations; Supplementary Table 2). The contribution of each prokaryotic phylum to the dissimilarity between poles was investigated using SIMPER analysis (Supplementary Table 3). The phyla with a significant contribution to the dissimilarity between the poles were less abundant groups including Chloroflexi and the WPS-2 group. To further investigate the distribution of ASVs and its differences between the poles, occupancy of each ASV across the cryoconite holes was plotted against mean relative abundance of that ASV for each polar region. Both the polar regions contain a “core group” of ASVs that are present in the majority of cryoconite holes and each contribute >1% of total abundance. However, the genera in these groups differ between the Arctic and Antarctic. Whilst both groups contain Microbacteriaceae, the remaining members are specific to the core group of each polar region. All core taxa contained in the Arctic and Antarctic groups are present in some proportion on both poles with the exception of Blastocatellaceae.

### Eukaryote Composition and Community Assembly

**Figure 6:**
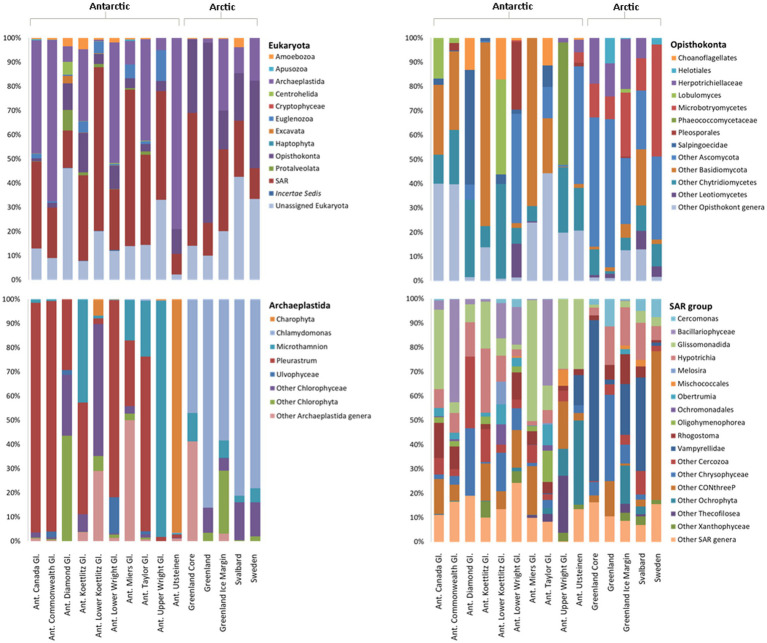
Relative abundance of microbial eukaryotes in Arctic and Antarctic cryoconite, averaged by glacier. Metazoa were excluded from these data. Top left: eukaryotic phyla. Phyla contributing to <1% of the total abundance are not included. Remaining panels: Relative abundance of genera within the top three most abundant phyla. Genera contributing to <1% of the total abundance are grouped as “Other.” Where a genus was unknown, lowest rank known is shown. Taxa were assigned according to the SILVA database. “Ant. Gl.” denotes Antarctic Glaciers.

Following filtering, 6,261 18S rRNA gene sequences were analysed from each Arctic and Antarctic cryoconite sample, which were assigned to a total of 11 eukaryotic phyla. Around 6,865 distinct 18S rRNA gene ASVs were found, 82 of which were shared between the Arctic and Antarctic. Metazoa accounted for 31% of 18S rRNA gene ASVs. Parachela tardigrades were the most abundant metazoans in the Arctic (64% of Arctic metazoan ASVs and 39% of Antarctic metazoan ASVs) and Adinetida rotifers were the most abundant metazoans in the Antarctic (36% of Arctic metazoan ASVs and 59% of Antarctic metazoan ASVs). The remaining metazoa recovered were Monogononta Ploimida and the platyhelminth Rhabdocoela Neodalyellida, both of which were only present in Antarctic cryoconite holes (contributing to 0.23 and 1.34% of Antarctic metazoan ASVs, respectively). Figure 6 shows microbial eukaryotes without metazoan 18S rRNA gene sequences. About 94% ASVs assigned to microbial eukaryotic phyla were assigned to the SAR group, Opisthokonta and Archaeplastida.

The 18S rRNA gene eukaryote taxonomic community structure showed less a clear divide between the Arctic and Antarctic cryoconite samples. However, when examining weighted UniFrac distances and abundance of taxa we observed variation of microbial eukaryote composition with polar region (Supplementary Figure 1). A much larger proportion of microbial Opisthokonts was found in the Arctic, 32% of assigned Arctic sequences belonged to the Opisthokonta compared to only 6% in the Antarctic. SIMPER analysis showed microbial Opisthokonts contributed significantly to the dissimilarity between poles (Supplementary Table 3). Several eukaryotic phyla present in the Antarctic were absent in Arctic cryoconites, but all of these phyla contribute less than 0.1% to the total number 18S rRNA gene sequences across both poles with exception for 18S rRNA gene sequences assigned to Euglenozoawhich were present in all Antarctic locations but the Upper Wright glacier. The Euglenozoa made up 2.5% of the total ASVs.

The most abundant eukaryotic phyla were Opisthokonta, Archaeplastida, and SAR group, which were investigated in further detail. The most striking difference between the Arctic and Antarctic compositions of the SAR group is that the Vampyrellidae, a predatory family of cercozoans, made up 32% of the Arctic SAR 18S rRNA gene sequences but less than 1% of Antarctic 18S rRNA gene sequences in Greenland and Svalbard. The sites in Sweden had 61% of its SAR 18S rRNA genes sequences belonging to CONthreeP ciliates. Bacillariophyceae (diatoms) represented 12% of Antarctic SAR sequences but 0.1% of Arctic sequences (an average of 4.75 ASVs assigned to Bacillariophyceae present, found in the Greenland Margin samples).

Around 89% of Archaeplastida were assigned to one of only four genera. Around 3% of the remaining sequences were unassigned Chlorophyta, 3% to unassigned Chlorophyceae, 3% to unassigned Uvlophyceae, and the remaining 18S rRNA gene sequences each contributed to >1% of the community composition. In the Archaeplastida, 18S rRNA gene sequence from the Arctic, 67% belonged to the snow algae genus *Chlamydomonas*, and 16% of 18S rRNA gene sequences were attributed to unassigned Chlorophyta. In contrast, the Antarctic cryoconite samples contained 0.1% *Chlamydomonas* 18S rRNA gene sequences. Around 52% belonged to *Pleurastrum*, 13% to *Microthamnion*, and 26% to an unassigned group of Charophyta. No Arctic 18S rRNA gene ASVs were assigned to *Pleurastrum* or Charophyta.

Of the 52 genera assigned to the Opisthokonta, only 11 appear in both the Arctic and Antarctic samples. Overall, 66% of Opisthokonta sequences belonged to the metazoa. The other metazoa contributed less than 1% to the total number 18S rRNA gene sequences. To better distinguish the microbial community, metazoa were excluded from the Opisthokonta in Figure 6. Of the microbial Opisthokonts, all samples were dominated by a group of unassigned Ascomycota fungi (39% of total microbial Opisthokont sequences). Microbotryomycetes (19% of Arctic and 1% of Antarctic Opisthokonta) and Herpotrichiellaceae (12% of Arctic and 2% of Antarctic microbial Opisthokonta) were also significant contributors to the Arctic microbial Opisthokonta, whereas Chytridiomycetes were more abundant in the Antarctic (19% of Antarctic sequences and 5% of Arctic sequences). All other assigned groups contributed to >4% of total microbial Opisthokont ASVs.

The mean number of different ASVS was 102 in the Arctic samples and 125 in the Antarctic samples (Supplementary Table 1). Alpha diversity and evenness of eukaryotes in the samples was further investigated using the Shannon diversity indices (Figure 4B). Similar to 16S rRNA gene communities, the lowest richness was in cryoconite from the Antarctic Lower Wright (2.30) and Upper Wright (3.32) glaciers. However, the highest values were from the Arctic: Svalbard cryoconite (5.94) and the Greenland margin sediment samples (5.87). The average values were 4.00 for the Antarctic and 5.02 for the Arctic sites. Only five of the total assigned 18S rRNA gene ASVs was present in more than one sample. These belonged to (by lowest rank assigned) *Monomastix minuta*, Herpotrichiellaceae, Microbotryomycetes, and Ascomycota.

## Discussion

### Differences in Community Composition Between Arctic and Antarctic Cryoconite Holes

**Figure 7:**
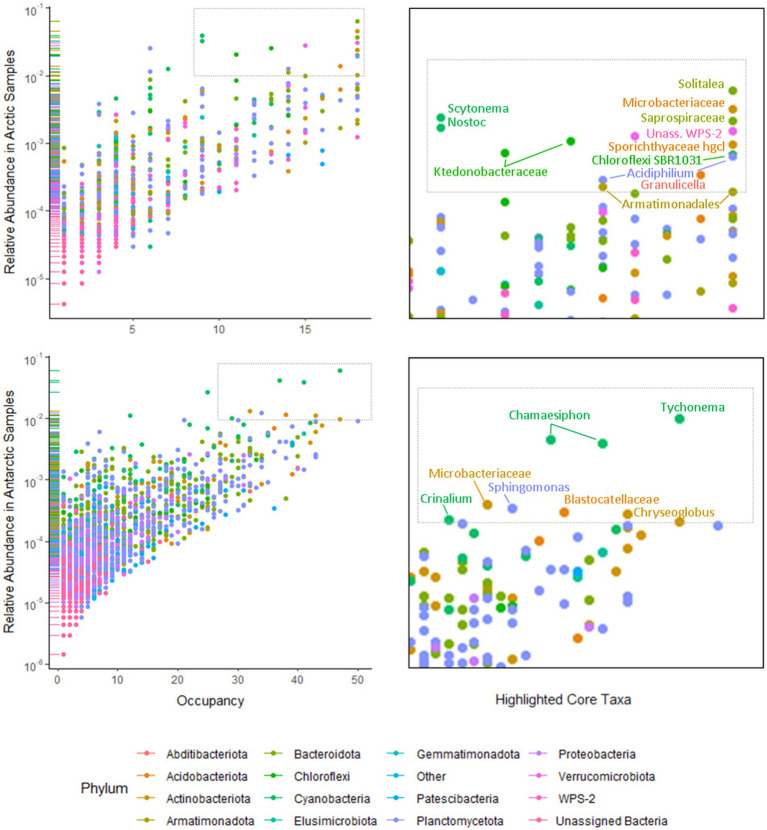
Occupancy of 16S rRNA ASVs in Arctic (upper left) and Antarctic (lower left) cryoconite holes by phyla. Phyla contributing to <1% of the total 16S rRNA gene sequences were grouped as “Other.” Taxa present in ≥75% of samples from that polar region that contributed to ≥1% of relative abundance are highlighted as core taxa (upper and lower right).

The use of Illumina sequencing enabled the examination of community composition across more locations and to a greater depth than previous comparisons of Arctic-Antarctic cryoconite microbial communities (e.g., Mueller et al., 2001; Cameron et al., 2012). The comparison of 16S and 18S rRNA gene communities shows that variation between poles was greater than between glaciers or individual cryoconite holes. The bacterial and archaea assemblages in particular clustered according to polar region (Figures 3, 5). Additionally, of the “core group” of ASVs that were both more abundant (contributing to >1% of total abundance) and present in the majority of cryoconite holes in each pole, only two genera were shared between the Arctic and Antarctic (Figure 7). Greater than 99% of eukaryotic ASVs were only present in one sample, illustrating that unlike the biogeography of bacteria in cryoconite, highly localised variation between cryoconite communities are predominant over regional and hemispheric differences. However, within each of the most abundant eukaryotic phyla (Archaeplastida, Opisthokonta, and the SAR group), there were genera contributing to a compositional divide between the cryoconite of the two polar regions. The results agree with findings by Cameron et al. (2012) that Arctic and Antarctic cryoconite holes harbour distinct bacterial and eukaryotic communities.

One possible explanation for this is that the glacier surface environments of the two poles have different physical characteristics governed by differences in temperature, radiation, and surrounding landscapes, and so may exhibit different selection pressures. For example, lower air and ice temperatures mean that many of the Antarctic cryoconite holes were closed (ice lidded), as is typical for the McMurdo Dry Valleys (Fountain et al., 2004). Most of these holes had a thick layer of sediment at the bottom (between 1cm and 10cm). By contrast, the Arctic cryoconite holes tend to be open and contain thinner, more aggregated sediment (<1cm thickness), which is often clustered into granules (Cook et al., 2016). The ice lid also results in lower levels of light reaching the cryoconite. These differences in habitat may create preferential conditions for some species, altering the community composition and selecting for particular species. Indeed, abiotic variability between cryoconite holes (e.g., pCO2, mineral availability, and temperature) has been shown to impact community structure (Edwards et al., 2011) and Antarctic cryoconite communities are adapted to low light availability (Bagshaw et al., 2016).

Limitations of transport and dispersal have also been shown to contribute to community differences (Telford et al., 2006). The Antarctic soil ecosystem, which is one important source of cryoconite matter, has limited connectivity to the airborne non-polar microbial pool (Pearce et al., 2009; Bottos et al., 2014; Archer et al., 2019). Similar dispersal limitation has been found in the Arctic soils through studies on Actinobacteria community assemblage (Eisenlord et al., 2012). Therefore, differences in community composition may not be solely caused by differing environments, but selection due to transport. There are also strong local winds in Antarctica and transport of biological material have been documented for the McMurdo Dry Valleys (Šabacká et al., 2012; Michaud et al., 2012), which may overshadow lower levels of biological material from long-range transport. If limitations of aeolian transport leads to selection, it follows that there should be segregation between the Arctic and Antarctic microbial communities. Through biogeographical analyses it may be possible for future studies to determine the contribution of these abiotic factors to community dissimilarity, although this is beyond the scope of our analysis. Co-correlation mapping between taxa and environmental variables, such as pCO2, mineral availability, temperature, and cryoconite hole physical parameters would be beneficial, as has been carried out previously in soil microorganisms (King et al., 2010). It would also be valuable to obtain transcriptomic data in addition to gene metabarcoding or genomic data to ascertain differences in active communities (Shakya et al., 2019). A comparison of active and legacy genes in cryoconite holes may also lend insight into which organisms successfully disperse within regions.

It is most likely that both geographical separation and the environment within the poles contribute the community differences we have found. Our 16S and 18S rRNA gene results show that a number of ASVs were present across all locations on one pole and absent on the other. The proportion of highly abundant groups such as Proteobacteria and Cyanobacteria vary between poles, but the significant differences include low abundance phyla: Chloroflexi and the WPS-2 supergroup (Supplementary Table 3). Surprisingly, the proportion of photosynthetic organisms was lower in the Arctic than the Antarctic samples (14% of Arctic 16S rRNA gene sequences, 32% of Antarctic 16S rRNA gene sequences, 20% of Arctic 18S rRNA gene sequences, and 49% of Antarctic 18S rRNA gene sequences). This may have implications for the overall autotrophy of the system. It should be noted that the present study uses ribosomal RNA gene sequences, which naturally cannot differentiate between living, dormant, or legacy sources of ribosomal RNA gene fragments (Blazewicz et al., 2013; Edwards et al., 2020). Such legacy genes may be stored inside cryoconite granules, which show stratification between a photoautotroph-rich exterior and the storage of degraded organic matter within their interiors (Takeuchi et al., 2001; Langford et al., 2010) and is potentially consistent with the enhanced accumulation of phylotypes within larger cryoconite granules (Uetake et al., 2016). By contrast, ribosomal RNA (cDNA) based analyses of communities in western Greenland return highly distinctive (potentially) active communities notable for the dominance of photoautotrophic bacterial lineages (Stibal et al., 2015; Gokul et al., 2019). It is therefore possible that differences in the retention and flushing of legacy DNA between cryoconite habitats may contribute to some difference in gene community composition; however, this is a factor that varies within the polar regions as well as between them.

Environmental differences and the limits of transport contribute to biogeographical clustering within glaciers (Edwards et al., 2011), and this is demonstrated in our data. Cryoconite communities within individual glaciers also tended to cluster, and so the differences between the glaciers could be viewed as the same contributing factors as the differences between poles on a smaller scale. In the Arctic and in Queen Maud Land in Antarctica, geographical clustering is stronger in the bacteria than eukaryotes (Cameron et al., 2012; Lutz et al., 2019), but curiously this result was not previously found in the McMurdo Dry Valleys (Cameron et al., 2012). Our results show clustering to be stronger in the bacteria across all locations on both poles, including the McMurdo Dry Valley sites.

The major phyla present (Actinobacteria, Bacteroidetes, Cyanobacteria, Proteobacteria, Archaeplastida, Opisthokonta, and the SAR group) are consistent with prior studies (Cameron et al., 2012; Kaczmarek et al., 2016) but there were some notable differences. *Mrakia*, a psychrophilic genus were absent in the Antarctic cryoconite and one of the more abundant opisthokont groups in the Arctic samples, despite having been discovered in the Antarctic (Xin and Zhou, 2007). In the eukaryotic microalgae, a higher proportion of *Chlamydomonas* and lower proportion of unknown Chloroccocales algae have been reported in Antarctic cryoconites (Christner et al., 2003; Sommers et al., 2018). Similarly, Bacillariophyceae (diatoms) were found in the Antarctic cryoconite as has been reported previously (Stanish et al., 2013) but, with the exception of one ASV recovered from the Greenland Margin, were absent in the Arctic cryoconite in contrast with previous findings from Greenland and Svalbard (Yallop et al., 2012; Vinšová et al., 2015). Although we found a clear split between the microalgae of the Arctic and Antarctic, it does not follow that those algae are only present on one pole. In light of previous studies, it is more likely that cryoconite holes became dominated by the residents of algal blooms flushed into those cryoconite holes in the season were collected (Yallop et al., 2012; Williamson et al., 2020). Sampling at other times of year and in other locations may yield a different algal community. In addition, 6% of the Archaeplastida could not be assigned to a genus or family (Figure 6), so could potentially belong to groups mentioned above or others that have been found in polar cryoconite but not detected here such as Zygnematophyceae (Vonnahme et al., 2016).

### 16S rRNA and 18S rRNA Gene High Throughput Sequencing Reveals High Taxonomic and Functional Diversity Across Both Poles

Through 16S and 18S rRNA gene sequencing we recovered a total of 35 phyla and superphyla; a considerably higher taxonomic diversity compared to previous studies that did not use next generation sequencing (Mueller et al., 2001; Christner et al., 2003; Mueller and Pollard, 2004; Porazinska et al., 2004; Hodson et al., 2010; Cameron et al., 2012). The most abundant phyla (16S rRNA gene: Bacteroidetes, Actinobacteria, Cyanobacteria, and Proteobacteria; 18S rRNA gene: SAR group, Archaeplastida, and Opisthokonta) were present in all samples, although the relative abundance and genera present varied. The average species richness of the Arctic was lower than that of the Antarctic, however, species richness varied widely between glaciers within each polar region. The Antarctic cryoconite assemblages showed more variation, both when measured by ASVs and by the Shannon diversity index (Supplementary Table 1). In contrast to the findings reported by Sommers et al., 2018, only a weak correlation was found between bacterial and eukaryotic diversity in cryoconite holes (*r*=4.1). The environmental conditions on individual glaciers, such as position within the valley, may contribute to variations in species richness (Stanish et al., 2013).

Bacterial and eukaryotic photoautotrophs were found across all locations and several mixotrophs were recovered, including the *Chlamydomonas* which dominate the Arctic eukaryotic algal population. We also detected chemotrophic bacteria such as *Thiobaccillus*, which plays an important part in sulphur cycling in subglacial environments (Harrold et al., 2015), and may have a role in sulphate reduction in the anoxic zone of cryoconite holes (Bagshaw et al., 2007; Poniecka et al., 2018). There is also a range of organisms predating and grazing on the community. The Vampyrellidae are likely microbial predators in the Arctic cryoconite habitats. Other heterotrophic and predatory groups such as the Ciliophora were found across both poles. Tardigrades and rotifers were found on glaciers with the exception of Sweden. Sweden may be outside the range of Arctic metazoa, though the microbial community is otherwise remarkably similar to that of the Svalbard cryoconite. The tardigrades and rotifers are likely to have been alive and active in the community, as these metazoans are commonly present in cryoconite communities (Porazinska et al., 2004; Zawierucha et al., 2021), and are key constituents of adjacent soil communities (Treonis et al., 1999; Virginia and Wall, 1999). Together these data suggest a complex trophic web within cryoconite holes across both poles, with metazoans as the top level grazer, microbial predation and heterotrophy, chemotrophy, diverse bacterial photoautotrophy, and microalgal assemblages but dominated by a small number of families.

Archaea were detected in very low relative abundances (<0.001% of 16S rRNA gene sequences) in the Arctic and Antarctic cryoconite holes. Other 16S rRNA gene sequencing surveys on cryoconite have found comparably low relative abundances of archaea (Lutz et al., 2015, 2017; Sommers et al., 2018) when using universal 16S rRNA gene primers (Caporaso et al., 2012). This may be due to these primers being less well suited for the amplification of the archaeal 16S rRNA gene (Parada et al., 2016). Investigations that have used specific primers designed for archaea detected more archaeal ASVs richness, though they are still minor component in comparison to the overall bacteria diversity recovered from cryoconite holes (Cameron et al., 2012; Weisleitner et al., 2020).

### Microbial Community Difference Between Glacial Environments

While cryoconite aggregate material can remain in a site for some years, the cryoconite holes may be flushed, buried, and otherwise deformed (Hodson et al., 2008; Bagshaw et al., 2012). In the Arctic sites this may happen more regularly, on a seasonal or sub seasonal timescale, whereas McMurdo Dry Valley cryoconite holes may not be completely flushed for several years (Foreman et al., 2007). Cryoconite is also washed towards the glacial margins over time, in the direction of melt. Previous studies have found retention of a local foundational community on glacier sites over time, but also selection based on highly localised environments (Edwards et al., 2011; Gokul et al., 2016; Segawa et al., 2017). As well as comparing supraglacial habitats between poles, we were also able to compare the communities of ice margin open cryoconite holes, mid-ice sheet open cryoconite holes, and ice covered cryoconite holes formed the previous year in Greenland to broaden our representation of Arctic cryoconite and examine the impact of local habitat within glacier sites. In Svalbard, it has been established that the microbial communities of cryoconite holes were distinct from those of the ice margins, with only a minority of phylotypes appearing in both habitats (Edwards et al., 2013). We confirmed that there is also a distinct difference between cryoconite communities found 60km onto the ice sheet and those within a few hundred metres of the ice margin in Greenland. There was significantly more variation between the types of Greenland samples (margin, ice core, and ice sheet surface) than within groups. The communities from cryoconite core samples were similar to the communities obtained from the surface Greenland cryoconite. The samples from Greenland core cryoconite and surface cryoconite formed two distinct clusters, but were considerably more similar to each other than to the other Arctic and Antarctic cryoconite communities (Supplementary Figure 2). This suggests consistency over the subsequent year as well as location in the Greenland cryoconite, and reinforces its distinction from the community present at the Greenland margin, which is likely influenced by uprafted subglacial debris (Knight et al., 2002). Mixing with a distinctive subglacial microbial community results in a higher abundance of methanogenic and sulphate reducing groups in margin samples when comparing to interior samples (Poniecka, 2020).

The Utsteinen region is situated in Queen Maud Land, an understudied region considerably far removed from the McMurdo Dry Valleys sites (Lutz et al., 2019). Despite the distance between the McMurdo Dry Valley and Utsteinen glaciers, the microbial community composition showed a high similarity. This suggests that there might be similar environmental drivers and sources for microbial communities in cryoconite holes across the Antarctic continent. Both regions are arid inland and glaciers will likely support closed-lidded cryoconite holes. They house recognisable hemisphere-specific cryoconite communities despite the limitations of aeolian transport across the Antarctic (Pearce et al., 2009). However, the Upper Wright Glacier samples bore a closer resemblance to the Arctic samples in several metrics and in some aspects, such the presence of Charophyta in several cryoconite holes, they were unique. There is no clear explanation for this. The Upper Wright sampling site was somewhat distinctive, as it is far from the sea and high altitude (950m) compared to the other Dry Valley samples. However, there is no certainty in the contributing factors to the Upper Wright’s distinct ecology.

## Conclusion

The use of 16S and 18S rRNA gene high throughput sequencing enabled a more comprehensive examination of taxonomic diversity of bacteria, archaea, and eukaryotes across Arctic and Antarctic cryoconite ecosystems than previous studies. We were able to resolve community composition to the family and often genus level, revealing a diverse community of microbes that contribute to a complex tropic web. Most significantly, it allowed for the direct comparison of microbial assemblages in cryoconite holes from both the Arctic and Antarctic. Our findings suggest that the Arctic and Antarctic cryoconite holes harbour distinct microbial communities, but the various biotic niches (grazer, predator, photoautotroph, and chemotroph) are filled in every location. The “core taxa,” which are numerous in both abundance and occupancy, share little similarity between the poles. The characteristics of the local environment and neighbouring habitats play a distinct role in the community composition. Therefore while cryoconite holes may be a global feature of glacier landscapes, they are inhabited by regionally distinct microbial communities.

## Data Availability Statement

The dataset presented in this study can be found at NCBI, accession project: PRJNA744712. https://www.ncbi.nlm.nih.gov/bioproject/744712.

## Author Contributions

JM, AJ, EB, and AE devised the project outline. JM conducted DNA extraction, sequencing, and analysis according to aims set by AJ. AJ, EB, and EP collected and provided cryoconite samples. JM wrote the resulting manuscript with contribution and approval from all authors. All authors contributed to the article and approved the submitted version.

## Funding

JM was funded by a UK Natural Environmental Research Council Great Western 4+ Doctoral Training Partnership, which also funded the DNA sequence analysis. McMurdo Dry Valley (MCM) samples were collected with the support of the US National Science Foundation MCM Long Term Ecological Research site. Greenland interior samples were obtained during the Black and Bloom fieldwork campaign,^1^ funded by UK NERC NE/M021025/1. Collection of the Utsteinen scoop samples was funded by the Baillet Latour Antarctica Fellowship (2016–2018) awarded to Lori Ziolkowski and by the Helmholtz Recruiting Initiative (award no: I-044-16-01) granted to Liane G. Benning.

## Conflict of Interest

The authors declare that the research was conducted in the absence of any commercial or financial relationships that could be construed as a potential conflict of interest.

## Publisher’s Note

All claims expressed in this article are solely those of the authors and do not necessarily represent those of their affiliated organizations, or those of the publisher, the editors and the reviewers. Any product that may be evaluated in this article, or claim that may be made by its manufacturer, is not guaranteed or endorsed by the publisher.

## Footnotes

^1^ https://blackandbloom.org/
