# Supplementary material for: Polar Cryoconite Associated Microbiota Is Dominated by Hemispheric Specialist Genera
Source: Front Microbiol. 2021 Nov 25;12:738451. doi: 10.3389/fmicb.2021.738451 (PMC8660574; doi:10.3389/fmicb.2021.738451)
Supplement: Supplementary file 1 [file Table_1.DOCX]

Supplementary Tables (1-3)

| 16s |  |  |  |  | 18s | |  |  |
| --- | --- | --- | --- | --- | --- | --- | --- | --- |
| Location | Average ASVs | Shannon | Simpson |  | Location | Average ASVs | Shannon | Simpson |
| Ant.Canada Gl. | 488.444 | 6.659 | 0.956 |  | Ant.Canada Gl. | 116.556 | 4.197 | 0.792 |
| Ant. Commonwealth Gl. | 615.111 | 7.152 | 0.967 |  | Ant. Commonwealth Gl. | 123.333 | 3.755 | 0.734 |
| Ant. Diamond Gl. | 226.500 | 5.557 | 0.950 |  | Ant. Diamond Gl. | 87.000 | 5.320 | 0.959 |
| Ant. Koettlitz Gl. | 271.833 | 5.624 | 0.932 |  | Ant. Koettlitz Gl. | 75.125 | 3.382 | 0.757 |
| Ant. Lower Koettlitz Gl. | 349.600 | 6.164 | 0.952 |  | Ant. Lower Koettlitz Gl. | 112.000 | 5.097 | 0.928 |
| Ant. Wright Gl. | 169.500 | 4.850 | 0.896 |  | Ant. Wright Gl. | 26.600 | 2.603 | 0.739 |
| Ant. Miers Gl. | 475.200 | 6.558 | 0.953 |  | Ant. Miers Gl. | 132.000 | 4.625 | 0.854 |
| Ant. Taylor Gl. | 325.250 | 6.057 | 0.946 |  | Ant. Taylor Gl. | 45.000 | 3.886 | 0.856 |
| Ant. Upper Wright Gl. | 114.000 | 4.762 | 0.914 |  | Ant. Upper Wright Gl. | 93.000 | 3.324 | 0.677 |
| Ant. Usteinen Scoop | 530.000 | 6.302 | 0.929 |  | Ant. Usteinen Scoop | 104.000 | 4.805 | 0.900 |
| Greenland Core | 136.667 | 5.112 | 0.944 |  | Greenland Core | 54.000 | 3.998 | 0.877 |
| Greenland | 141.333 | 5.349 | 0.958 |  | Greenland | 118.000 | 5.055 | 0.937 |
| Greenland Ice Margin | 333.500 | 6.295 | 0.963 |  | Greenland Ice Margin | 158.750 | 5.672 | 0.957 |
| Svalbard | 273.600 | 5.966 | 0.962 |  | Svalbard | 111.429 | 5.891 | 0.969 |
| Sweden | 230.333 | 6.141 | 0.972 |  | Sweden | 91.667 | 4.685 | 0.926 |

Supplementary table 1: Alpha diversity microorganisms in cryoconite holes as measured by average number of 16S and 18S ASVs, and the Shannon and Simpson indices. Metazoa were removed from the 18S sample set.

Supplementary table 2: ANOSIM analysis of 16S and 18S ASVs in between polar regions and individual glaciers across the Arctic and Antarctic. Shown over all taxa, and most abundant phyla individually. Significance levels of <0.05 are marked with “*”.

|  | Polar Region |  | Location |  |
| --- | --- | --- | --- | --- |
| Group | R Value | Significance | R Value | Significance |
| All 16S | 0.723 | 1.00E-04* | 0.7175 | 1.00E-04* |
| Cyanobacteria | 0.4583 | 1.00E-04* | 0.6017 | 1.00E-04* |
| Actinobacteria | 0.5552 | 1.00E-04* | 0.7378 | 1.00E-04* |
| Proteobacteria | 0.6611 | 1.00E-04* | 0.7530 | 1.00E-04* |
| Bacteroidetes | 0.2418 | 1.00E-04* | 0.2461 | 1.00E-04* |
| All 18S | 0.02559 | 0.0859 | 0.1171 | 1.00E-04* |
| SAR Group | 0 | 1 | 0 | 1 |
| Archaeplastida | 0 | 1 | 0 | 1 |
| Opisthokonta | 0.1603 | 0.0134* | 0.5454 | 1.00E-04* |

Supplementary Table 3: Similarity percentages (SIMPER) of phyla contributions to Arctic and Antarctic communities. Significance codes: 0 ‘***’ 0.001 ‘**’ 0.01 ‘*’ 0.05 ‘.’ 0.1 ‘ ’.

|  | Average | SD | Ratio | Average (Antarctic) | Average (Arctic) | Cumulative Sum | p-value |  |
| --- | --- | --- | --- | --- | --- | --- | --- | --- |
| Bacteria and Archaea |  |  |  |  |  |  |  |  |
| Cyanobacteria | 8.08E-02 | 6.19E-02 | 1.3047 | 3.21E+03 | 1.16E+03 | 0.2007 | 0.19802 |  |
| Proteobacteria | 4.76E-02 | 3.57E-02 | 1.3356 | 2.51E+03 | 2.79E+03 | 0.319 | 0.732673 |  |
| Chloroflexi | 3.09E-02 | 3.24E-02 | 0.9546 | 1.88E+02 | 9.32E+02 | 0.3959 | 0.019802 | * |
| WPS-2 | 3.07E-02 | 2.22E-02 | 1.381 | 9.93E+01 | 8.63E+02 | 0.4721 | 0.009901 | ** |
| Bacteroidetes | 2.77E-02 | 2.05E-02 | 1.351 | 2.18E+03 | 2.41E+03 | 0.5409 | 0.663366 |  |
| Patescibacteria | 2.69E-02 | 2.44E-02 | 1.1054 | 5.33E+02 | 6.24E+02 | 0.6079 | 0.415842 |  |
| Actinobacteria | 2.41E-02 | 1.69E-02 | 1.4266 | 1.20E+03 | 1.15E+03 | 0.6677 | 0.722772 |  |
| Armatimonadetes | 2.24E-02 | 1.15E-02 | 1.9455 | 3.26E+02 | 8.11E+02 | 0.7232 | 0.059406 | . |
| Acidobacteria | 2.15E-02 | 1.31E-02 | 1.6494 | 7.08E+02 | 8.10E+02 | 0.7767 | 0.920792 |  |
| Abditibacteriota | 2.13E-02 | 1.99E-02 | 1.0715 | 4.66E+02 | 3.99E+02 | 0.8297 | 0.891089 |  |
| Unassigned Bacteria | 1.97E-02 | 1.82E-02 | 1.0853 | 4.79E+02 | 4.02E+02 | 0.8787 | 1 |  |
| Planctomycetes | 1.80E-02 | 1.07E-02 | 1.6801 | 5.00E+02 | 4.02E+02 | 0.9235 | 0.138614 |  |
| Verrucomicrobia | 1.28E-02 | 9.14E-03 | 1.4027 | 3.66E+02 | 3.29E+01 | 0.9554 | 0.049505 | * |
| Gemmatimonadetes | 7.23E-03 | 3.48E-03 | 2.0766 | 2.31E+02 | 4.19E+01 | 0.9733 | 0.009901 | ** |
| Firmicutes | 5.02E-03 | 8.27E-03 | 0.6064 | 2.02E+01 | 1.29E+02 | 0.9858 | 0.168317 |  |
| Deinococcota | 4.21E-03 | 3.43E-03 | 1.2283 | 1.19E+02 | 6.04E+01 | 0.9962 | 0.732673 |  |
| Fibrobacterota | 6.87E-04 | 6.64E-04 | 1.0339 | 1.11E+01 | 1.13E+01 | 0.9979 | 0.09901 | . |
| Caldisericota | 5.52E-04 | 8.21E-04 | 0.6719 | 9.10E-01 | 1.42E+01 | 0.9993 | 0.108911 |  |
| Sumerlaeota | 1.67E-04 | 2.96E-04 | 0.5656 | 4.39E+00 | 1.67E-02 | 0.9997 | 0.50495 |  |
| Elusimicrobiota | 5.65E-05 | 1.13E-04 | 0.499 | 1.50E+00 | 0.00E+00 | 0.9999 | 0.306931 |  |
| Nitrospirota | 4.80E-05 | 1.29E-04 | 0.3706 | 1.25E+00 | 1.67E-02 | 1 | 0.792079 |  |
| Dependentiae | 6.70E-06 | 1.30E-05 | 0.5146 | 1.68E-01 | 1.67E-02 | 1 | 0.891089 |  |
| Archaea | 3.62E-06 | 7.70E-06 | 0.4694 | 9.44E-02 | 0.00E+00 | 1 | 0.910891 |  |
| Hydrogenedentes | 8.52E-07 | 2.58E-06 | 0.33 | 2.22E-02 | 0.00E+00 | 1 | 1 |  |
| Eukarya |  |  |  |  |  |  |  |  |
| Archaeplastida | 1.62E-01 | 0.149004 | 1.089 | 1312.368 | 512.28 | 0.2844 | 0.861386 |  |
| Opisthokonta | 1.40E-01 | 0.124563 | 1.1237 | 222.351 | 1128.8 | 0.5297 | 0.009901 | ** |
| SAR | 1.35E-01 | 0.109929 | 1.2265 | 1382.504 | 932.07 | 0.766 | 0.891089 |  |
| Unassigned Eukarya | 9.81E-02 | 0.083798 | 1.1709 | 658.329 | 921.48 | 0.938 | 0.60396 |  |
| Euglenozoa | 1.69E-02 | 0.024459 | 0.6902 | 137.745 | 0 | 0.9676 | 0.584158 |  |
| Amoebozoa | 7.36E-03 | 0.007764 | 0.9473 | 31.522 | 45.63 | 0.9805 | 0.138614 |  |
| Protalveolata | 5.77E-03 | 0.010936 | 0.5279 | 36.452 | 0 | 0.9906 | 0.70297 |  |
| Centrohelida | 2.65E-03 | 0.006717 | 0.3942 | 15.802 | 0.2 | 0.9952 | 0.673267 |  |
| Excavata | 1.56E-03 | 0.003938 | 0.3965 | 9.429 | 0 | 0.998 | 0.712871 |  |
| Apusozoa. | 5.69E-04 | 0.001065 | 0.5347 | 3.44 | 0 | 0.999 | 0.722772 |  |
| Incertae Sedis | 4.96E-04 | 0.000886 | 0.5596 | 3.895 | 0 | 0.9998 | 0.633663 |  |
| Haptophyta | 5.62E-05 | 0.000173 | 0.3257 | 0.5 | 0 | 0.9999 | 0.683168 |  |
| Cryptophyceae | 3.60E-05 | 0.000111 | 0.3257 | 0.32 | 0 | 1 | 0.683168 |  |
