# Supplementary material for: Polar Cryoconite Associated Microbiota Is Dominated by Hemispheric Specialist Genera
Source: Front Microbiol. 2021 Nov 25;12:738451. doi: 10.3389/fmicb.2021.738451 (PMC8660574; doi:10.3389/fmicb.2021.738451)
Supplement: Supplementary file 2 [file Data_Sheet_1.PDF]

Supplementary Figures (1-2)

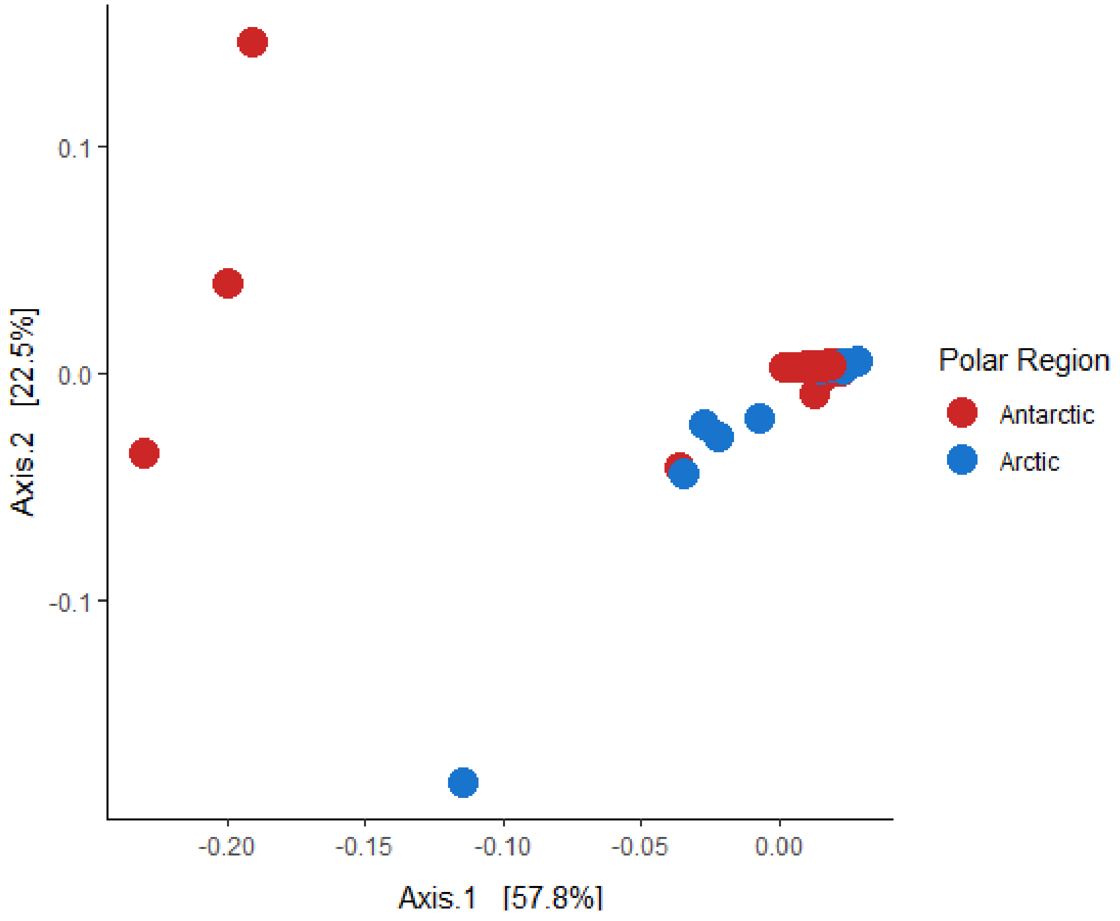

Supplementary Figure 1: Weighted Unifrac distances of eukaryotic ASVs in Arctic and Antarctic cryoconite grouped by polar region. Metazoa were excluded.

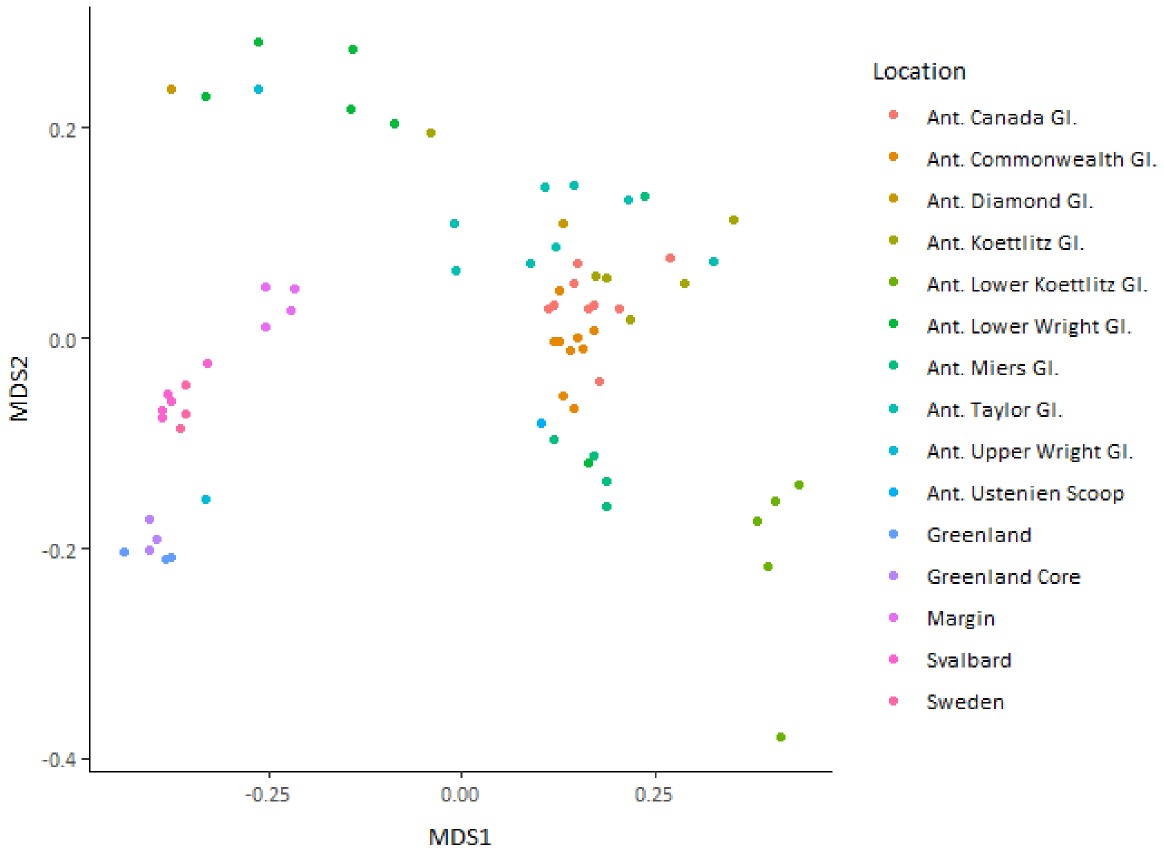

Supplementary figure 2: Bray-Curtis dissimilarity of 16S rRNA gene ASVs found in each cryoconite and visualised by non-metric multidimensional scaling (NMDS) ordination. Cryoconite holes are grouped by glacier.
